# High platelet-to-lymphocyte ratio is associated with poor prognosis in patients with unresectable intrahepatic cholangiocarcinoma receiving gemcitabine plus cisplatin

**DOI:** 10.1186/s12885-020-07390-3

**Published:** 2020-09-23

**Authors:** Gunn Huh, Ji Kon Ryu, Jung Won Chun, Joo Seong Kim, Namyoung Park, In Rae Cho, Woo Hyun Paik, Sang Hyub Lee, Yong-Tae Kim

**Affiliations:** Department of Internal Medicine, Liver Research Institute, Seoul National University Hospital, Seoul National University College of Medicine, 101 Daehak-ro, Jongno-gu, Seoul, 110-744 South Korea

**Keywords:** Intrahepatic cholangiocarcinoma, Platelet-to-lymphocyte ratio, Overall survival

## Abstract

**Background:**

Several systemic inflammatory response (SIR) markers, including platelet-to-lymphocyte ratio (PLR), neutrophil-to-lymphocyte ratio (NLR), lymphocyte-to-monocyte ratio (LMR), and albumin-to-globulin ratio (AGR), have emerged as prognostic markers in various cancers. The aim of this study was to explore the impact of SIR markers on the survival outcomes of unresectable intrahepatic cholangiocarcinoma (IHC) patients.

**Methods:**

Patients with histologically confirmed, unresectable IHC treated with gemcitabine plus cisplatin (GP) chemotherapy in a single tertiary hospital from 2012 to 2016 were retrospectively reviewed. Progression-free survival (PFS) and overall survival (OS) were determined using unadjusted Kaplan-Meier and adjusted Cox-proportional-hazards analysis. Time-dependent receiver operating characteristic (ROC) analysis was performed to compare the performance of the SIR markers in predicting OS.

**Results:**

A total of 137 patients received a median of six cycles (interquartile range [IQR], 3–11) of GP chemotherapy with a median observation time of 9.9 months (range, 1.8–54.7 months). The median PFS and OS of all patients were 7.8 months and 9.9 months, respectively. Among the SIR markers, high PLR (> 148) and high NLR (> 5) were associated with a short PFS (Hazard ratio [HR] 1.828, *P* = 0.006; HR 1.738, *P* = 0.030, respectively) and short OS (HR 2.332, *P* < 0.001; HR 2.273, *P* < 0.001, respectively). Low LMR (< 3.5) and low AGR (< 1.2) were associated with a short OS (HR 2.423, *P* < 0.001; HR 1.768, *P* = 0.002, respectively). In multivariable cox-regression analysis, high PLR (HR 1.766, *P* = 0.009) and distant lymph node (LN) metastasis (HR 2.085, *P* = 0.001) were associated with a short PFS. High PLR (HR 1.856, *P* = 0.002) was an independent predictor of a short OS, along with distant LN metastasis (HR 1.929; *P* < 0.001), low LMR (HR 1.691; *P* = 0.041), and low level of serum albumin (< 3.5 g/dL) (HR 1.632; *P* = 0.043). Time-dependent ROC analysis revealed that the area under the curve of PLR for predicting overall survival was greater than that of NLR, LMR, and AGR at most time points.

**Conclusions:**

High PLR was an independent prognostic factor of a short PFS and OS in patients with unresectable IHC receiving GP chemotherapy.

## Background

Intrahepatic cholangiocarcinoma (IHC) is cancer that originates from epithelial cells of the intrahepatic bile duct, which accounts for approximately 10% of all cholangiocarcinomas [1, 2]. IHC is a rare disease, though its prevalence varies enormously according to geographic regions. The incidence rates seem to be increasing globally. Surgical resection is the only curative treatment; however, only a minority of patients present with resectable disease. The prognosis for IHC is poor, and it remains a challenge to identify prognostic biomarkers to stratify patients and determine the optimal therapy.

Inflammation is a hallmark of cancer, and the inflammatory response plays an important role in cancer development and progression [3]. Several systemic inflammatory response (SIR) markers, such as platelet-to-lymphocyte ratio (PLR), neutrophil-to-lymphocyte ratio (NLR), lymphocyte-to-monocyte ratio (LMR), and albumin-to-globulin ratio (AGR), have been studied and recognized as prognostic factors in various cancers. Chronic inflammation is a key predisposing factor in the development of biliary tract cancer (BTC) [4], and there are several studies to evaluate the prognostic impact of SIR markers in BTC. However, most studies focused on the preoperative values of these markers in patients with resectable disease. To the best of our knowledge, there have not been any studies that have evaluated the prognostic impact of PLR, NLR, LMR, and AGR together in unresectable IHC patients receiving systemic chemotherapy.

The aim of the present study was to explore the prognostic value of these SIR markers in patients with unresectable IHC receiving first-line chemotherapy.

## Methods

### Study subjects

The medical records of patients diagnosed with unresectable IHC at Seoul National University Hospital from January 1st, 2012 through December 31st, 2016 were retrospectively reviewed. All adults aged 20 years or older with histologically diagnosed IHC, who received gemcitabine plus cisplatin (GP) chemotherapy, were included in the study. Patients who received GP chemotherapy for only one cycle or received best supportive care or locoregional therapy (e.g., transarterial chemoembolization or radiation therapy) were excluded. Patients with a history of concomitant malignancy or rheumatic disease within 5 years or missing laboratory values were also excluded from the study.

### Data collection and definitions

Demographic and clinical data were collected, including age, sex, body mass index, Eastern Cooperative Oncology Group performance status (ECOG PS), history of chronic hepatitis B or C or hepatolithiasis, presence of cirrhosis, Charlson comorbidity index, presence of concomitant malignancies or rheumatic disease, history of previous biliary drainage, presence of regional or distant lymph node (LN) metastasis, and site of metastasis. Regional LNs were defined according to the 8th edition of the AJCC staging manual [5]. Baseline laboratory values in venous blood, including complete blood cell count, liver function test, and CA 19–9 levels, were obtained within a week before the initiation of GP chemotherapy in patients without evidence of active infection.

PLR and NLR were calculated as the absolute platelet and absolute neutrophil count divided by the absolute lymphocyte count, respectively. LMR was calculated as the absolute lymphocyte count divided by the absolute monocyte count. AGR was the level of serum albumin divided by the serum globulin level. Because the optimal cut-off values for PLR, NLR, LMR, and AGR have not been established, maximally selected log-rank statistic by Hothorn and Lausen was used to determine the optimal cut-off values that represent the maximum difference in overall survival between groups [6]. R package ‘maxstat’ (https://CRAN.R-project.org/package=maxstat) was used for this analysis and adjusted *p*-values were calculated by the approximation based on an improved Bonferroni inequality with an alpha error of 0.05. Patients were stratified by the optimal values, and the clinical characteristics were compared across groups.

Data on survival outcomes were collected. Progression-free survival (PFS) was defined as the time from chemotherapy initiation until either disease progression or death due to any cause. Overall survival (OS) was the time from chemotherapy initiation until death or the last follow-up. Tumor response was assessed every 6 to 9 weeks according to the Response Evaluation Criteria in Solid Tumors (RECIST), version 1.1 [7]. The response rate was calculated as the proportion of patients with complete response or partial response. The disease control rate was defined as the proportion of patients with complete response, partial response, or stable disease. A minimum time interval of 6 weeks was required for patients to be considered evaluable for disease control.

### Statistical analysis

Data were presented as whole numbers with percentages for categorical variables and median with interquartile range (IQR) or mean ± standard deviation for continuous variables. The Chi-square and Mann-Whitney U tests were used to compare the categorical and continuous variables, respectively. Correlation among SIR markers were determined by the Spearman rank correlation coefficients and Pearson correlation coefficient. Estimates of the median follow-up period was calculated by the reverse Kaplan-Meier method [8]. Estimates of PFS and OS were calculated using the Kaplan-Meir method. The log-rank test was used to compare survival outcomes between the groups. All variables with a univariate *P* < 0.100 were subjected to multivariable analysis using the Cox proportional hazards model with backward elimination. The variance inflation factor was estimated to assess the multicollinearity of the final model. Considering the collinearity between SIR markers, we conducted further multivariable analyses to build separate models. This time, each SIR marker was assessed separately in different multivariable models. All reported *P*-values are two-sided, and *P* < 0.05 was considered significant. Time-dependent receiver operating characteristic (ROC) curves were created to assess the performance of PLR, NLR, LMR, and AGR in predicting OS. Time-dependent ROC curves were estimated using the ‘timeROC’ R package. All statistical analyses were performed using the RStudio version 3.44 statistical software package.

### Ethical standards

This study was approved by the Institutional Review Board of Seoul National University Hospital (IRB No. H-1705-120-855) and conducted in conformity with the Declaration of Helsinki.

## Results

### Clinical characteristics of the study population

**Fig. 1:**
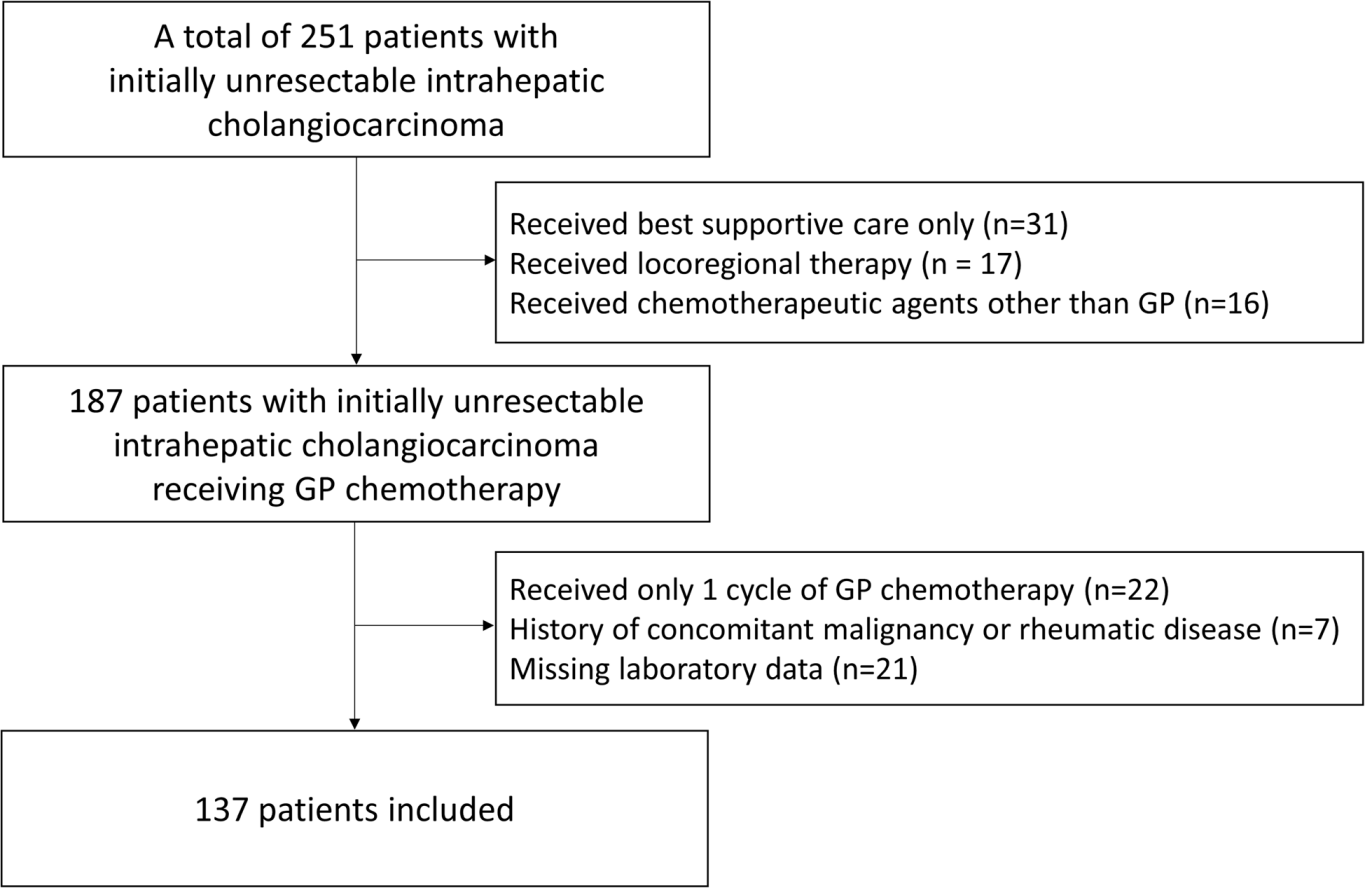
Flow chart of patient enrollment

A total of 251 patients were diagnosed with unresectable IHC at Seoul National University Hospital from January 1st, 2012 through December 31st, 2016. Of these 251 patients, 31 patients who received best supportive care only, 17 patients who received locoregional therapy, and 16 patients who received chemotherapeutic agents other than GP were excluded from the study. Among the remaining 187 patients who received GP chemotherapy, 22 patients received only one cycle of GP chemotherapy, seven patients had a history of concomitant malignancy or rheumatic disease, and 21 patients had missing laboratory data. These 50 patients were also excluded from the study. The remaining 137 patients were included in the study (Fig. 1).

**Fig. 2:**
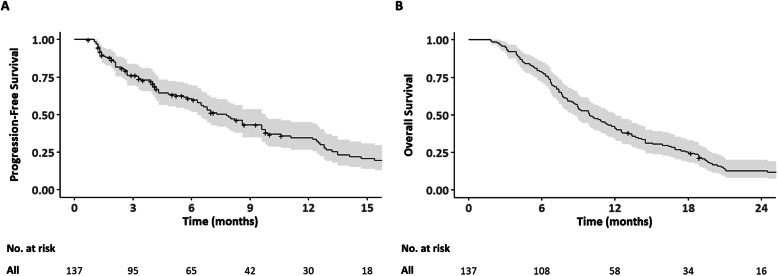
Progression-free survival (2**a**) and overall survival (2**b**) of all patients

The clinical features of the 137 patients are summarized in Table 1. The median follow-up period by the reverse Kaplan-Meier estimates was 35.4 months (95% CI, 34.9 months – not reached). The median observation time for all patients was 9.9 months (range, 1.8–54.7 months). The median number of GP chemotherapy cycles patients received was 6 cycles (IQR, 3–11 cycles). The median PFS was 7.8 months (95% CI, 6.3–9.8 months), and the median OS was 9.9 months (95% CI, 8.6–12.0 months) (Fig. 2). Among the 135 patients (98.5%) with measurable disease, the best overall responses included a partial response in 23 patients (17.0%), stable disease in 81 patients (60.0%), progressive disease in 27 patients (20.0%), and not assessable in four patients (3.0%), resulting in an overall response rate of 17.0% and disease control rate of 77.0%. Three patients (2.2%) with initial metastatic IHC underwent conversion surgery after GP chemotherapy with R0 resection achieved in two of these patients. Second-line chemotherapy after GP treatment failure was given to 71 patients (52.6%) and included fluoropyrimidine-based combination therapy (e.g. FOLFIRI [5-fluorouracil and irinotecan], iFAM [infusional 5-fluorouracil, doxorubicin, and mitomycin-C], XP [capecitabine and cisplatin]) for 45 patients (32.8%), fluoropyrimidine monotherapy for 17 patients (12.4%), and clinical trials for nine patients (6.6%). Palliative radiation therapy was administered in 13 patients (9.5%) and site of radiation included bone metastases for 11 patients (8.0%), portal vein tumor thrombosis for one patient (0.7%), and brain metastases for one patient (0.7%). None of the patients received transarterial chemoembolization or radioembolization.

**Table 1:** Baseline characteristics of all patients (*N* = 137)

Variable	No. (%) or median (IQR)
Age, yr	64 (57, 72)
Sex	
Male	83 (60.6%)
Female	54 (39.4%)
BMI	23.4 (22.0, 25.3)
ECOG Performance status	
0	64 (46.7%)
≥ 1	73 (53.3%)
Chronic hepatitis B	21 (15.3%)
Chronic hepatitis C	4 (2.9%)
Hepatolithiasis	17 (12.4%)
Liver cirrhosis	15 (10.9%)
Child-Pugh class A	12 (8.8%)
Child-Pugh class B	3 (2.2%)
Diabetes mellitus	25 (18.2%)
Charlson Commorbidity Index	
0	87 (63.5%)
≥ 1	50 (36.5%)
Biliary drainage	15 (10.9%)
Tumor size, cm	7.0 (5.0, 9.9)
Major vascular invasion	89 (65.0%)
Hilar invasion	16 (11.7%)
Liver metastasis	55 (40.1%)
Extrahepatic organ metastasis	75 (54.7%)
Peritoneum	35 (25.5%)
Lung	33 (24.1%)
Bone	25 (18.2%)
Distant lymph node metastasis	90 (65.7%)
Number of metastatic sites	
0	36 (26.3%)
1	59 (43.1%)
≥ 2	42 (30.7%)
Baseline laboratory findings	
White blood cell count, cells/μL	8060 (6700, 9440)
Neutrophil count, cells/μL	5606 (4126, 6751)
Lymphocyte count, cells/μL	1612 (1346, 2029)
Monocyte count, cells/μL	633 (509, 801)
Hemoglobin, g/dL	12.6 ± 1.7
Platelet count, 10^3^ cells/μL	237 (185, 281)
Albumin, g/dL	4.0 (3.6, 4.2)
Globulin, g/dL	3.2 (3.0, 3.6)
Total bilirubin, mg/dL	0.7 (0.5, 0.9)
ALP, IU/L	139 (98, 245)
AST, IU/L	34 (24, 51)
ALT, IU/L	26 (17, 44)
CA 19–9, U/mL	266 (27, 4280)
Platelet-to-lymphocyte ratio	133 (110, 186)
Neutrophil-to-lymphocyte ratio	3.4 (2.3, 4.4)
Lymphocyte-to-monocyte ratio	2.5 (2.1, 3.2)
Albumin-to-globulin ratio	1.2 ± 0.3

Abbreviations: *IQR* Interquartile range; *BMI* Body mass index; *ECOG* Eastern Cooperative Oncology Group; *ALP* Alkaline phosphatase; *AST* Aspartate aminotransferase; *ALT* Alanine aminotransferase; *CA 19–9* Carbohydrate antigen 19–9;

Data regarding hemoglobin and albumin-to-globulin ratio are presented as mean ± standard deviation

### Relationships between SIR markers

There was a strong correlation between PLR, NLR, and LMR as follows: PLR and NLR (Spearman’s rho = 0.61, *P* < 0.001), PLR and LMR (Spearman’s rho = − 0.46, *P* < 0.001), and NLR and LMR (Spearman’s rho = − 0.67, *P* < 0.001). AGR was associated with LMR (Pearson correlation coefficient = 0.21, *P* = 0.016), but there was no significant association with PLR (*P* = 0.514) or NLR (*P* = 0.358) (Supplementary Figure 1).

### Comparisons between groups according to optimal cut-off values

The optimal cut-off values for the SIR markers were 148 for PLR (*P* < 0.001), 5.0 for NLR (*P* = 0.020), 3.5 for LMR (*P* < 0.001), and 1.2 for AGR (*P* = 0.025) (Supplementary Figure 2). Patients were stratified into groups according to these values. The high PLR group (*N* = 63) had significantly lower albumin levels than the low PLR group (*N* = 74) (*P* = 0.030) (Table 2). There were no other significant differences in the clinical characteristics between the high and the low PLR groups. The clinical characteristics of patients, according to NLR, LMR, and AGR, are summarized in Supplementary Table 1. Patients with high NLR (*N* = 28), low LMR (*N* = 110), and low AGR (*N* = 72) had lower albumin levels compared to those with low NLR (*N* = 109), high LMR (*N* = 27), and high AGR (*N* = 65) (*P* = 0.011, *P* = 0.012, and *P* < 0.001, respectively). The high NLR and low AGR groups had a higher proportion of poor performance status (ECOG PS ≥ 1) than the low NLR and high AGR groups (*P* = 0.018 and *P* = 0.035, respectively). The high LMR and low AGR groups had higher number of metastatic sites than the low LMR and high AGR groups (*P* = 0.001 and *P* = 0.031, respectively).The low AGR group had a higher proportion of patients with distant LN metastasis compared to the high AGR group (*P* < 0.001).

**Table 2:** Clinical characteristics of patients according to PLR

Variable	PLR ≤148 (N = 74)	PLR > 148 (N = 63)	*P* value
Age, yr	64 (58, 73)	64 (54, 69)	0.247
Sex			1.000
Male	45 (60.8%)	38 (60.3%)	
Female	29 (39.2%)	25 (39.7%)	
BMI	23.7 (22.2, 25.7)	23.0 (22.0, 24.5)	0.119
ECOG Performance status			1.000
0	35 (47.3%)	29 (46.0%)	
≥ 1	39 (52.7%)	34 (54.0%)	
Liver cirrhosis	10 (13.5%)	5 (7.9%)	0.443
Diabetes mellitus	15 (20.3%)	10 (15.9%)	0.658
Charlson Commorbidity Index			0.595
0	45 (60.8%)	42 (66.7%)	
≥ 1	29 (39.2%)	21 (33.3%)	
Biliary drainage	6 (8.1%)	9 (14.3%)	0.379
Tumor size, cm	7.2 (5.4, 10.0)	7.0 (4.5, 9.6)	0.246
Major vascular invasion	53 (71.6%)	36 (57.1%)	0.112
Hilar invasion	9 (12.2%)	7 (11.1%)	1.000
Liver metastasis	27 (36.5%)	28 (44.4%)	0.440
Extrahepatic organ metastasis	38 (51.4%)	37 (58.7%)	0.489
Distant lymph node metastasis	47 (63.5%)	43 (68.3%)	0.688
Number of metastatic sites			0.090
0	10 (13.5%)	2 (3.2%)	
1	27 (36.5%)	23 (36.5%)	
≥ 2	37 (50.0%)	38 (60.3%)	
Baseline laboratory findings			
Neutrophil count, cells/μL	5288 (3768, 6403)	5792 (4663, 7231)	0.063
Lymphocyte count, cells/μL	1935 (1547, 2271)	1377 (1119, 1623)	< 0.001
Monocyte count, cells/μL	637 (509, 808)	629 (506, 756)	0.837
Platelet count, 10^3^ cells/μL	199 (163, 255)	261 (224, 303)	< 0.001
Total bilirubin, mg/dL	0.7 (0.6, 0.9)	0.7 (0.5, 0.9)	0.979
ALP, IU/L	129 (93, 225)	164 (115, 259)	0.074
Albumin, g/dL	4.0 (3.7, 4.2)	3.8 (3.5, 4.1)	0.030
CA 19–9, U/mL	652.5 (44.6, 4830.0)	183.4 (16.1, 2955.0)	0.211

Abbreviations: *PLR* Platelet-to-lymphocyte ratio; *BMI* Body mass index; *ECOG* Eastern Cooperative Oncology Group; *ALP* Alkaline phosphatase; *CA 19–9* Carbohydrate antigen 19–9

Data are presented as no. (%) or median (interquartile range)

### Association of SIR markers and survival outcomes

**Fig. 3:**
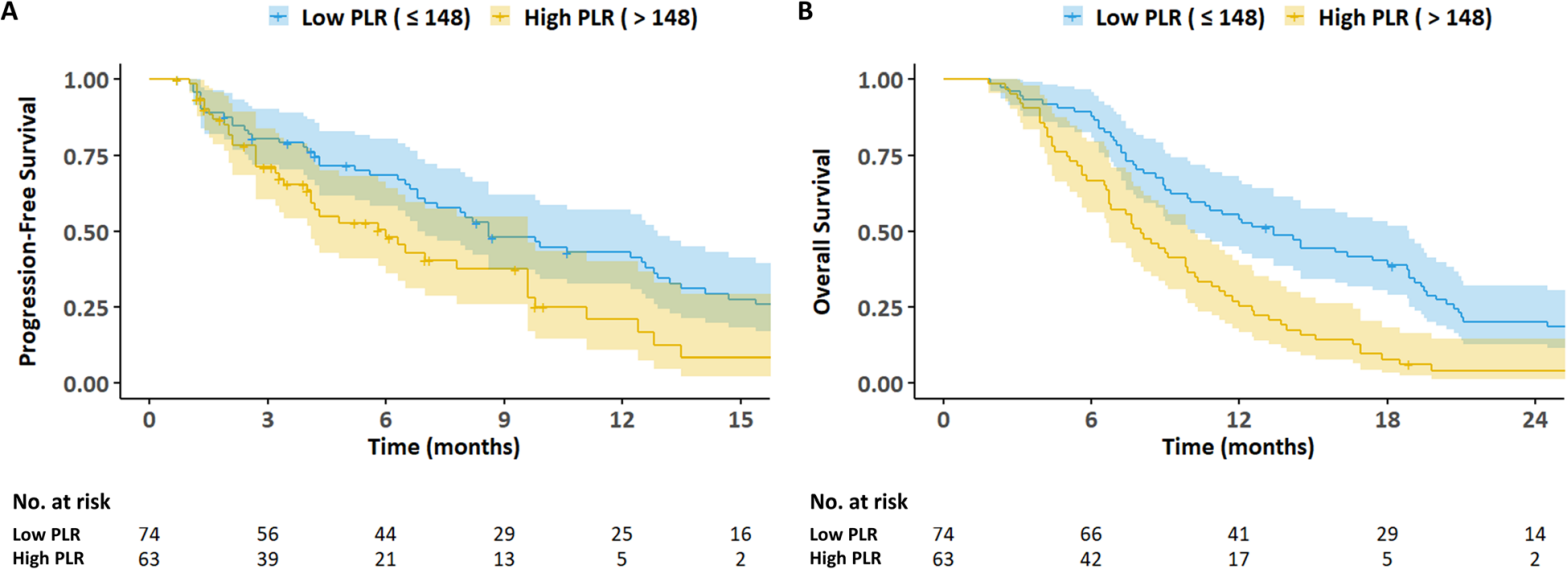
Progression-free survival (3**a**) and overall survival (3**b**) according to PLR. **a**: *P* = 0.005, **b**: *P* < 0.001 (log-rank test)

**Fig. 4:**
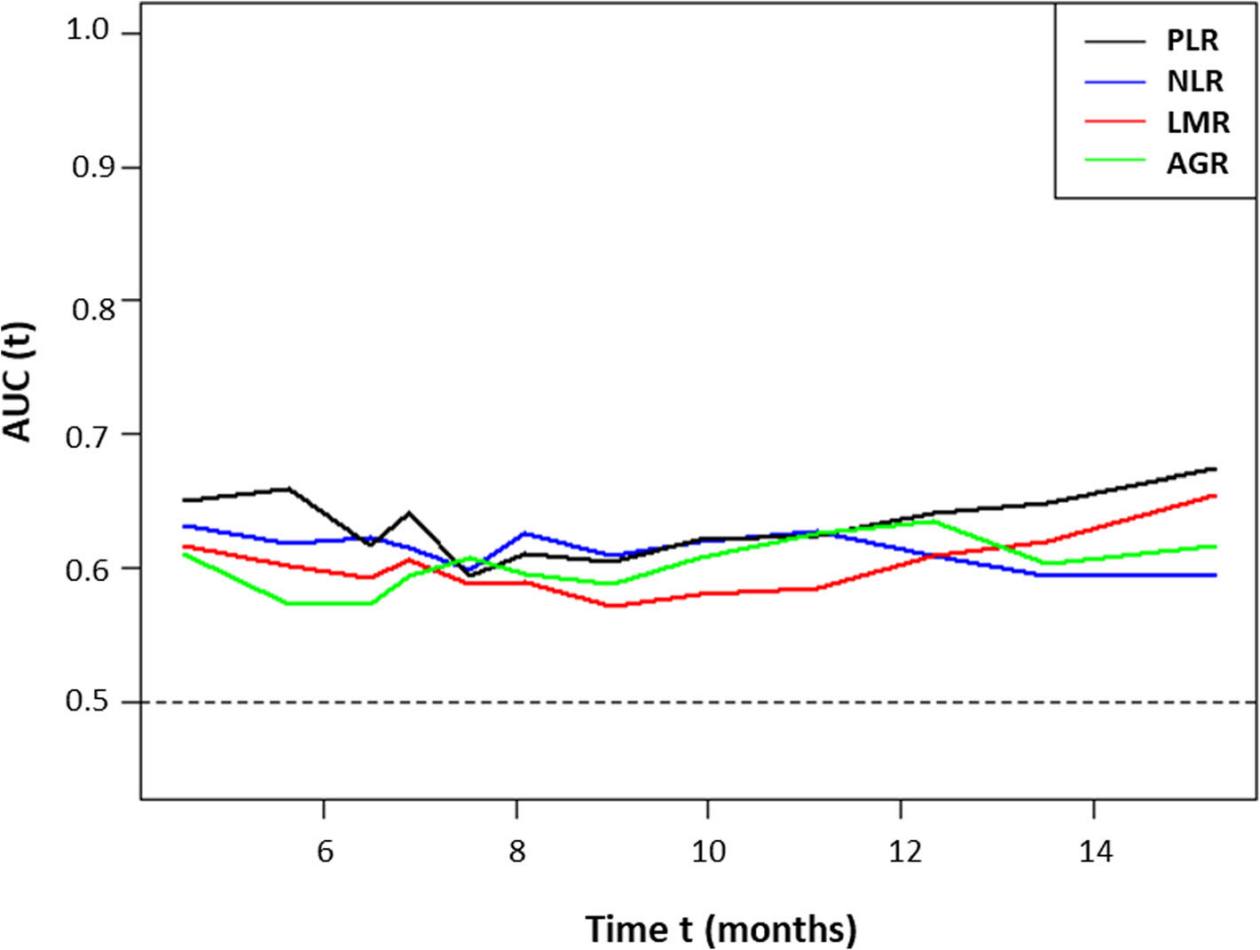
Time-dependent ROC analysis for PLR, NLR, and LMR according to overall survival

The median PFS was significantly different between the high and low PLR groups (6.0 vs. 8.6 months; *P* = 0.006) (Fig. 3a). A significant difference was also observed in the median OS for these groups (8.0 vs. 13.4 months; *P* < 0.001) (Fig. 3b). Similarly, the high and low NLR groups had significantly different median PFS (4.0 vs. 8.6 months; *P* = 0.030) and OS (6.7 vs. 11.7 months; *P* < 0.001). The low LMR group had a significantly shorter OS compared to the high LMR group (9.1 vs. 19.1 months; *P* < 0.001); however, the difference between the median PFS of these two groups was not statistically significant (6.8 vs. 9.9 months; *P* = 0.087). Similarly, the low AGR group had a significantly shorter OS compared to the high AGR group (8.7 vs. 13.2 months; *P* = 0.002), but the difference in the median PFS between these groups was not statistically significant (6.5 vs. 9.6 months; *P* = 0.368). The time-dependent ROC analysis revealed that the area under the curve of PLR for predicting overall survival was greater than that of NLR, LMR, and AGR at most time points (Fig. 4).

### Predictive factors for PFS

The results of the univariate and multivariable analyses for PFS are shown in Table 3. Multivariable analysis, which was performed using variables with a univariate *P* < 0.100, showed that high PLR (> 148) and distant LN metastasis were associated with a short PFS (Hazard ratio [HR] 1.766 [95% CI, 1.155–2.703], *P* = 0.009; HR 2.085 [95% CI, 1.329–3.272], *P* = 0.001, respectively). When SIR markers were assessed separately in multivariable models, high PLR (> 148) was associated with a short PFS (HR 1.766, *P* = 0.009), while high NLR (> 5) and low LMR (< 3.5) were not (HR 1.638 [95% CI, 0.995–2.696], *P* = 0.052; HR 1.583 [95% CI, 0.963–2.603], *P* = 0.070, respectively). (Supplementary Table 2).

**Table 3:** Univariable and multivariable Cox proportional hazard analysis of factors associated with progression free survival

	Univariable		Multivariable	
	HR (95% CI)	***P***	HR (95% CI)	***P***
Age		0.148		
≤ 65	1			
> 65	0.735 (0.484–1.116)			
Sex		0.149		
Male	0.797 (0.489–1.115)			
Female	1			
ECOG PS		0.518		
0	1			
≥ 1	1.139 (0.767–1.692)			
Charlson Commorbidity index		0.697		
0	1			
≥ 1	1.084 (0.722–1.626)			
Chronic hepatitis B		0.155		
No	1			
Yes	1.497 (0.934–2.320)			
Chronic hepatitis C		0.612		
No	1			
Yes	1.297 (0.475–3.547)			
Liver cirrhosis		0.157		
No	1			
Yes	1.551 (0.844–2.851)			
Biliary drainage		0.184		
No	1			
Yes	0.593 (0.275–1.281)			
Tumor size		0.394		
≤ 7 cm	1			
> 7 cm	0.838 (0.558, 1.258)			
Vascular invasion		0.379		
No	1			
Yes	0.830 (0.548, 1.257)			
Hilar invasion		0.181		
No	1			
Yes	0.610 (0.296, 1.258)			
Liver metastasis		0.472		
No	1			
Yes	1.157 (0.777–1.723)			
Extrahepatic organ metastasis		0.280		
No	1			
Yes	1.246 (0.836–1.856)			
Distant lymph node metastasis		**0.001**		**0.001**
No	1		1	
Yes	2.135 (1.361–3.348)		2.085 (1.329–3.272)	
Number of metastatic sites				
0	1			
1	2.567 (1.064–6.194)	**0.036**		
≥ 2	3.422 (1.467–7.985)	**0.004**		
Total bilirubin		0.336		
≤ 1.5 x ULN	1			
> 1.5 x ULN	0.611 (0.224–1.666)			
Alkaline phosphatase		0.765		
≤ 1.5 x ULN	1			
> 1.5 x ULN	1.066 (0.703–1.616)			
Albumin		0.050		
< 3.5 g/dL	1.725 (1.001–2.975)			
≥ 3.5 g/dL	1			
CA 19–9		0.818		
≤ 37 U/mL	1			
> 37 U/mL	0.955 (0.643–1.417)			
PLR		**0.006**		**0.009**
≤ 148	1		1	
> 148	1.828 (1.193–2.800)		1.766 (1.155–2.703)	
NLR		**0.030**		
≤ 5	1			
> 5	1.738 (1.056–2.859)			
LMR		0.087		
< 3.5	1.538 (0.939–2.520)			
≥ 3.5	1			
AGR		0.368		
< 1.2	1.200 (0.807–1.786)			
≥ 1.2	1			

Abbreviations: *ECOG* Eastern Cooperative Oncology Group; *CA 19–9* Carbohydrate antigen 19–9; *PLR* Platelet-to-lymphocyte ratio; *NLR* Neutrophil-to-lymphocyte ratio; *LMR*, lymphocyte-to-monocyte ratio; *AGR* Albumin-to-globulin ratio

### Predictive factors for OS

The results of the univariate and multivariate analyses for OS are shown in Table 4. Multivariable analysis showed that high PLR (> 148) (HR, 1.856 [95% CI, 1.266–2.723]; *P* = 0.002), low LMR (< 3.5) (HR 1.691 [95% CI, 1.023–2.797]; *P* = 0.041), distant LN metastasis (HR 1.929 [95% CI, 1.305–2.851]; *P* < 0.001), and low level of serum albumin (< 3.5 g/dL) (HR, 1.632 [95% CI, 1.017–2.618]; *P* = 0.043) were independent predictive factors of a short OS, after adjusting for alkaline phosphatase and ECOG PS. The highest variance inflation factor was 1.13, suggesting no significant multicollinearity between the variables in the final model. When SIR markers were assessed separately in multivariable models, high PLR (> 148), high NLR (> 5), low LMR (< 3.5), respectively remained as an independent predictor of short OS (HR 2.182 [95% CI, 1.512–3.150], *P* < 0.001; HR 1.714 [95% CI, 1.063–2.762], *P* = 0.027; HR 2.199 [95% CI, 1.367–3.538], *P* = 0.001, respectively) (Supplementary Table 3).

**Table 4:** Univariable and multivariable Cox proportional hazard analysis of factors associated with overall survival

	Univariable		Multivariable	
	HR	***P***	HR	***P***
Age		0.512		
≤ 65	1			
> 65	1.127 (0.788–1.610)			
Sex		0.452		
Male	0.872 (0.610–1.246)			
Female	1			
ECOG PS		0.090		0.092
0	1		1	
≥ 1	1.355 (0.953–1.926)		1.372 (0.949–1.984)	
Charlson Commorbidity index		0.730		
0	1			
≥ 1	1.066 (0.742–1.530)			
Chronic hepatitis B		0.246		
No	1			
Yes	1.328 (0.822–2.144)			
Chronic hepatitis C		0.736		
No	1			
Yes	1.187 (0.437–3.230)			
Liver cirrhosis		0.134		
No	1			
Yes	1.531 (0.877–2.673)			
Biliary drainage		0.884		
No	1			
Yes	1.043 (0.597–1.821)			
Tumor size		0.207		
≤ 7 cm	1			
> 7 cm	0.799 (0.564, 1.132)			
Vascular invasion		0.346		
No	1			
Yes	0.841 (0.587, 1.206)			
Hilar invasion		0.752		
No	1			
Yes	0.914 (0.524, 1.596)			
Liver metastasis		0.686		
No	1			
Yes	0.930 (0.654–1.322)			
Extrahepatic organ metastasis		0.108		
No	1			
Yes	1.332 (0.939–1.889)			
Distant lymph node metastasis		**< 0.001**		**< 0.001**
No	1		1	
Yes	1.906 (1.309–2.775)		1.929 (1.305–2.851)	
Number of metastatic sites				
0	1			
1	2.753 (1.292–5.864)	**0.009**		
≥ 2	2.987 (1.434–6.221)	**0.003**		
Total bilirubin		0.604		
≤ 1.5 x ULN	1			
> 1.5 x ULN	1.209 (0.590–2.480)			
Alkaline phosphatase		**0.008**		0.125
≤ 1.5 x ULN	1		1	
> 1.5 x ULN	1.632 (1.138–2.340)		1.337 (0.923–1.939)	
Albumin		**0.001**		**0.043**
< 3.5 g/dL	2.268 (1.424–3.610)		1.632 (1.017–2.618)	
≥ 3.5 g/dL	1		1	
CA 19–9		0.530		
≤ 37 U/mL	1			
> 37 U/mL	1.118 (0.790–1.583)			
PLR		**< 0.001**		**0.002**
≤ 148	1		1	
> 148	2.332 (1.610–3.378)		1.856 (1.266–2.723)	
NLR		**< 0.001**		
≤ 5	1			
> 5	2.273 (1.471–3.512)			
LMR		**< 0.001**		**0.041**
< 3.5	2.423 (1.516–3.875)		1.691 (1.023–2.797)	
≥ 3.5	1		1	
AGR		**0.002**		
< 1.2	1.768 (1.236–2.528)			
≥ 1.2	1			

Abbreviations: *ECOG* Eastern Cooperative Oncology Group; *CA 19–9* Carbohydrate antigen 19–9; *PLR* Platelet-to-lymphocyte ratio; *NLR* Neutrophil-to-lymphocyte ratio; *LMR* Lymphocyte-to-monocyte ratio; *AGR* Albumin-to-globulin ratio

## Discussion

This study is the first to evaluate the association of PLR, NLR, LMR, and AGR together with PFS and OS in patients with unresectable IHC. We found that high PLR (> 148) was an independent prognostic factor of a short PFS and OS in patients with unresectable IHC receiving GP chemotherapy. Time-dependent ROC analysis revealed that the area under the curve of PLR for predicting overall survival was greater than that of the other inflammatory markers (i.e., NLR, LMR, or AGR) at most time points.

SIR markers, including PLR, NLR, LMR, and AGR, have been investigated for an association with the prognosis of BTC. Studies on the association of PLR and survival outcomes in BTC patients are relatively scarce and mostly only included patients undergoing surgical resection [9] [10]. Cho et al. [11] reported that high NLR (≥3.8) and high PLR (≥121) were independent predictors of a short OS for patients with advanced BTC, including IHC, extrahepatic cholangiocarcinoma, gallbladder cancer, and ampulla of Vater cancer. In our study, high PLR (> 148), but not high NLR (> 5), was an independent predictor of a short PFS and OS in patients with advanced IHC. The discrepancies might be due to the different study populations of the two studies. In the previous study, the distribution of PLR varied according to tumour origin (*P* = 0.003). There has been accumulating evidence indicating extreme molecular and biological heterogeneity in BTC, according to the anatomical location of the tumour [12, 13]. Therefore, it is reasonable to assume that the SIR markers might have different prognostic impacts depending on the cancer type. Another study that included only patients with advanced IHC reported that high NLR (> 2.8) and high PLR (> 128.3) were associated with a short PFS or OS by univariate analysis, but not multivariable analysis [14]. The reason for this difference is not clear; however, the different cut-off values might be one explanation. Because optimal cut-off values for PLR and NLR have not been established, the previous study chose median values as cut-off values, while we selected cut-off points that showed maximal differences in OS. In addition, the previous study did not investigate the impact of LMR but explored the association of the modified Glasgow Prognostic Score, which is based on the level of albumin and C-reactive protein. There have not been any studies that have evaluated LMR in advanced IHC. However, in advanced gallbladder cancer, our group recently reported that a high monocyte to lymphocyte ratio (> 0.24) and high PLR (> 108) were associated with poor survival [15].

The exact mechanisms by which PLR predicts survival outcomes in cancer patients are not clear. However, there are plausible mechanisms to explain how thrombocytosis and lymphopenia are associated with poor prognosis in cancer patients. The association between platelets and cancer has been studied since 1865 when Trousseau first described thrombosis in gastric cancer [16]. Tumour cells are known to activate platelets and stimulate platelet aggregation. Activated platelets can mediate cancer cell growth and angiogenesis [17]. They can also directly protect circulating tumour cells from natural killer cell-mediated lysis, which promotes metastatic dissemination [18]. In experimental mouse models, the induction of thrombocytopenia reduced the rate of metastasis, while reconstitution with human platelets increased the number of metastases in vivo [19, 20]. Clinical studies have shown that thrombocytosis is associated with poor prognosis in several cancers, including breast, lung, colon, gastric, and ovarian cancer [21]. The lymphocyte is a key mediator of immunosurveillance and antitumor immunity [22]. A low number of lymphocytes could be responsible for a weak immune response against cancer cells.

Data on the association of PLR and OS according to the stage of BTC are insufficient; however, studies conducted in other solid cancers suggest that the significance of PLR is greater for metastatic disease than for early-stage disease [23, 24]. Furthermore, PLR values were greater in metastatic disease than in early-stage disease. In our study, PLR was not associated with the level of CA 19–9 or organ/lymph node metastasis and the number of metastatic sites. These findings might be due to the small number of patients with advanced disease that were included in this study. Further studies comprising a large number of patients with different stages and tumour burden are needed to investigate the association of PLR and other clinical variables in IHC.

Not surprisingly, there were strong correlations between SIR markers. PLR was positively associated with NLR and negatively associated with LMR. This SIR marker was also negatively associated with albumin, but there was no significant association with AGR. Albumin has been classically known to reflect nutritional status; however, recent studies have shown that the level of albumin is decreased during inflammation, regardless of nutritional status [25]. Globulin plays an important role in immunity and inflammation. Because levels of both albumin and globulin are easily influenced by factors, such as dehydration or oedema, AGR has been suggested and explored as a prognostic factor in several cancers [26]. In the present study, the serum albumin levels and AGR were significantly associated with distant LN metastasis, which was the strongest independent predictive factor of PFS and OS. Low AGR did not remain as an independent prognostic factor of OS after multivariable analysis, which might be due to its association with the presence of distant LN metastasis and ECOG PS.

There are several limitations to the current study. First, it was a single-centre, retrospective study, and the number of patients comprising the study population was small. Due to the rarity of IHC, the number of IHC patients is limited in a single-centre design. However, multicentre studies have a pitfall that stems from different blood processing techniques between laboratories. Second, the current study included only patients who received at least two cycles of GP. We believed that this approach could minimize the potential confounding effect that might result from different anticancer therapies. Thus, the results may not be generalizable to other populations, including patients receiving non-GP chemotherapy, concurrent chemoradiation therapy, locoregional therapy, or best supportive care only. Third, the dichotomous cut-off value of the SIR markers might be arbitrary. Currently, optimal cut-off values for the SIR markers have not been established, and they vary between studies. We chose to use dichotomized cut-offs because previous studies utilizing such cut-offs have shown a better association between PLR and overall survival than those with three risk categories [23]. The optimal cut-off values in the present study, which were determined using a statistical method, were similar to those of the previous studies [26–29]. Nevertheless, they warrant further validation in large, prospective studies since outcome-oriented approach to select cut-off value might result in overfitting.

## Conclusions

High PLR (> 148) might be a useful prognostic factor of a short PFS and OS in patients with unresectable IHC who received first-line chemotherapy. Low LMR (< 3.5) was an independent prognostic factor of a short OS. Low AGR was associated with the presence of distant LN metastasis, which was an independent prognostic factor of a short PFS and OS. Further studies are needed to validate the prognostic impact of SIR markers in unresectable IHC.

## Supplementary information

**Additional file 1: Supplementary Figure 1.** Relationship between PLR, NLR, LMR, and AGR.

**Additional file 2: Supplementary Figure 2.** Determination of optimal cut-off values using maximally selected log-rank statistics. A: optimal cut-off for PLR was 148 (*P* < 0.001), B: optimal cut-off for NLR was 5.0 (*P* = 0.020), C: optimal cut-off for LMR was 3.5 (*P* < 0.001), D: optimal cut-off for AGR was 1.2 (*P* = 0.025).

**Additional file 3: Supplementary Table 1.** Clinical characteristics of patients according to NLR, LMR, and AGR. **Supplementary Table 2.** Multivariable Cox regression analysis of factors associated with progression-free survival (SIR marker-specific model). **Supplementary Table 3.** Multivariable Cox regression analysis of factors associated with overall survival (SIR marker-specific model).

## Data Availability

The datasets used and/or analysed during the current study are available from the corresponding author on reasonable request.

## Abbreviations

SIR: Systemic inflammatory response
PLR: Platelet-to-lymphocyte ratio
NLR: Neutrophil-to-lymphocyte ratio
LMR: Lymphocyte-to-monocyte ratio
AGR: Albumin-to-globulin ratio
IHC: Intrahepatic cholangiocarcinoma
GP: Gemcitabine plus cisplatin
PFS: Progression-free survival
OS: Overall survival
ROC: Receiver operating characteristic
IQR: Interquartile range
HR: Hazard ratio
LN: Lymph node
BTC: Biliary tract cancer
ECOG PS: Eastern cooperative oncology group performance status
