# Supplementary figures and images for: High platelet-to-lymphocyte ratio is associated with poor prognosis in patients with unresectable intrahepatic cholangiocarcinoma receiving gemcitabine plus cisplatin
Source: BMC Cancer. 2020 Sep 23;20:907. doi: 10.1186/s12885-020-07390-3 (PMC7510111; doi:10.1186/s12885-020-07390-3)

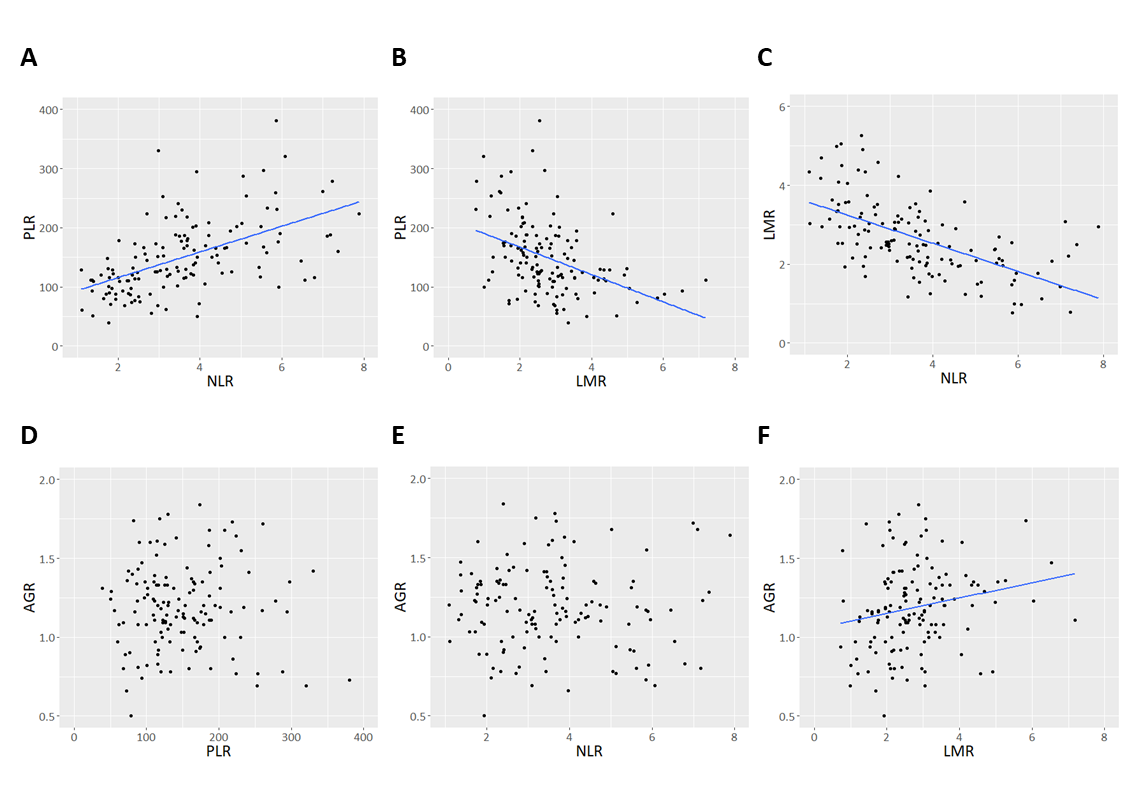

Supplement: Supplementary file 1 — Additional file 1: Supplementary Figure 1. Relationship between PLR, NLR, LMR, and AGR. [file 12885_2020_7390_MOESM1_ESM.tif]

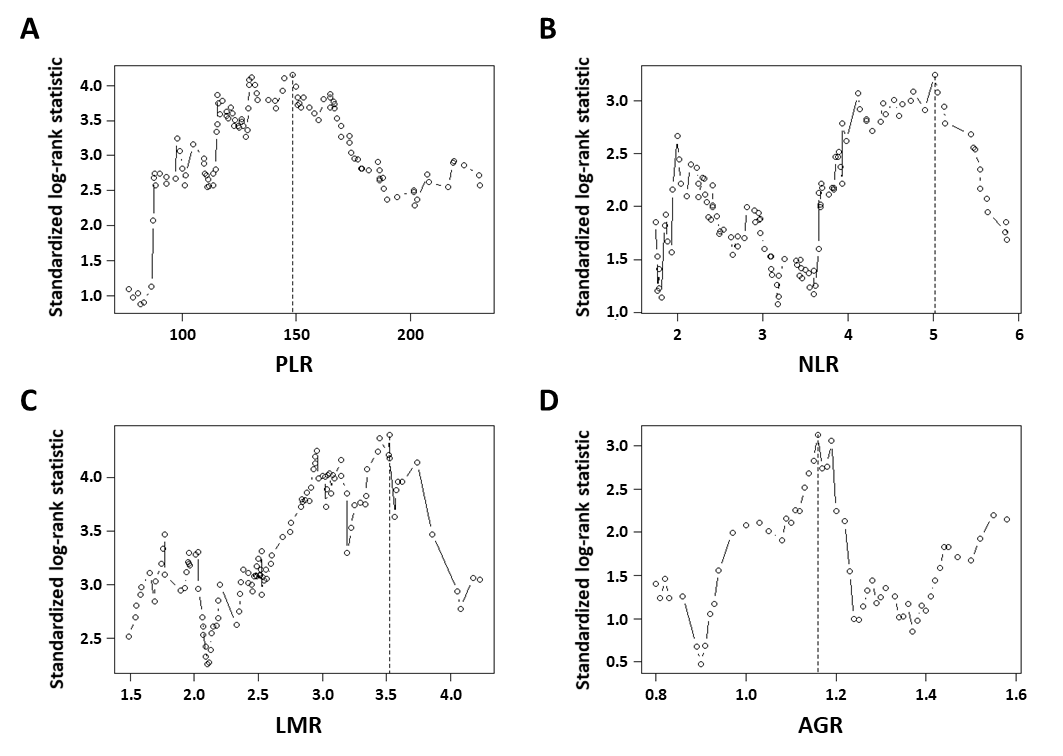

Supplement: Supplementary file 2 — Additional file 2: Supplementary Figure 2. Determination of optimal cut-off values using maximally selected log-rank statistics. A: optimal cut-off for PLR was 148 (P < 0.001), B: optimal cut-off for NLR was 5.0 (P = 0.020), C: optimal cut-off for LMR was 3.5 (P < 0.001), D: optimal cut-off for AGR was 1.2 (P = 0.025). [file 12885_2020_7390_MOESM2_ESM.tif]
