# Supplementary material for: High platelet-to-lymphocyte ratio is associated with poor prognosis in patients with unresectable intrahepatic cholangiocarcinoma receiving gemcitabine plus cisplatin
Source: BMC Cancer. 2020 Sep 23;20:907. doi: 10.1186/s12885-020-07390-3 (PMC7510111; doi:10.1186/s12885-020-07390-3)
Supplement: Supplementary file 3 — Additional file 3: Supplementary Table 1. Clinical characteristics of patients according to NLR, LMR, and AGR. Supplementary Table 2. Multivariable Cox regression analysis of factors associated with progression-free survival (SIR marker-specific model). Supplementary Table 3. Multivariable Cox regression analysis of factors associated with overall survival (SIR marker-specific model). [file 12885_2020_7390_MOESM3_ESM.docx]

| **Supplementary Table 1. Clinical characteristics of patients according to NLR, LMR, and AGR** | | | | | | | | | |
| --- | --- | --- | --- | --- | --- | --- | --- | --- | --- |
| **Variable** | **NLR** | | | **LMR** | | | **AGR** | | |
|  | **≤ 5 (N=109)** | **>5 (N=28)** | ***P* value** | **≥3.5 (N=27)** | **<3.5 (N=110)** | ***P* value** | **≥1.2 (N=65)** | **<1.2 (N=72)** | ***P* value** |
| Age, yr | 64 (58, 72) | 60 (50, 73) | 0.192 | 62 (58, 69) | 64 (55, 72) | 0.766 | 63 (57, 68) | 64 (56, 73) | 0.349 |
| Sex |  |  | 0.285 |  |  | 1.000 |  |  | 0.510 |
| Male | 69 (63.3%) | 14 (50.0%) |  | 16 (59.3%) | 67 (60.9%) |  | 37 (56.9%) | 12 (16.7%) |  |
| Female | 40 (36.7%) | 14 (50.0%) |  | 11 (40.7%) | 43 (39.1%) |  | 28 (46.2%) | 60 (83.3%) |  |
| BMI |  |  |  | 23.4 (22.3, 25.9) | 23.3 (22.0, 25.3) | 0.368 | 23.4 (22.2, 25.4) | 23.3 (21.9, 25.0) | 0.687 |
| ECOG PS |  |  | 0.018 |  |  | 0.417 |  |  | 0.035 |
| 0 | 57 (52.3%) | 7 (25.0%) |  | 15 (55.6%) | 49 (44.5%) |  | 37 (56.9%) | 27 (37.5%) |  |
| ≥1 | 52 (47.7%) | 21 (75.0%) |  | 12 (44.4%) | 61 (55.5%) |  | 28 (43.1%) | 45 (62.5%) |  |
| CCI |  |  | 0.449 |  |  | 0.875 |  |  | 0.133 |
| 0 | 67 (61.5%) | 20 (71.4%) |  | 18 (66.7%) | 69 (62.7%) |  | 46 (70.8%) | 41 (56.9%) |  |
| ≥1 | 42 (38.5%) | 8 (28.6%) |  | 9 (33.3%) | 41 (37.3%) |  | 19 (29.2%) | 31 (43.1%) |  |
| Diabetes mellitus | 22 (20.5%) | 3 (10.7%) | 0.377 | 5 (18.5%) | 20 (18.2%) | 1.000 | 10 (15.4%) | 15 (20.8%) | 0.546 |
| Liver cirrhosis | 12 (11.0%) | 3 (10.7%) | 1.000 | 2 (7.4%) | 13 (11.8%) | 0.754 | 3 (4.6%) | 12 (16.7%) | 0.048 |
| Biliary drainage | 11 (10.1%) | 4 (14.3%) | 0.768 | 5 (18.5%) | 10 (9.1%) | 0.288 | 3 (4.6%) | 12 (16.7%) | 0.048 |
| Tumor size, cm | 6.9 (5.0, 9.8) | 7.4 (5.0, 10.5) | 0.448 | 5.6 (4.6, 8.2) | 7.2 (5.1, 10.0) | 0.056 | 7.0 (5.0, 10.0) | 7.2 (4.9, 9.7) | 0.733 |
| Vascular invasion | 71 (65.1%) | 18 (64.3%) | 1.000 | 18 (66.7%) | 71 (64.5%) | 1.000 | 41 (63.1%) | 48 (66.7%) | 0.795 |
| Hilar invasion | 12 (11.0%) | 4 (14.3%) | 0.879 | 3 (11.1%) | 13 (11.8%) | 1.000 | 9 (13.8%) | 7 (9.7%) | 0.628 |
| Liver metastasis | 44 (40.4%) | 11 (39.3%) | 1.000 | 9 (33.3%) | 46 (41.8%) | 0.557 | 27 (41.5%) | 28 (38.9%) | 0.888 |
| Extrahepatic organ  metastasis | 57 (52.3%) | 18 (64.3%) | 0.355 | 12 (44.4%) | 63 (57.3%) | 0.325 | 37 (56.9%) | 38 (52.8%) | 0.753 |
| Distant lymph node  metastasis | 70 (64.2%) | 20 (71.4%) | 0.622 | 15 (55.6%) | 75 (68.2%) | 0.311 | 30 (46.2%) | 60 (83.3%) | <0.001 |
| Number of metastatic sites |  |  | 0.248 |  |  | 0.001 |  |  | 0.031 |
| 0 | 11 (10.1%) | 1 (3.6%) |  | 7 (25.9%) | 5 (4.5%) |  | 10 (15.4%) | 2 (2.8%) |  |
| 1 | 42 (38.5%) | 8 (28.6%) |  | 6 (22.2%) | 44 (40.0%) |  | 23 (35.4%) | 27 (37.5%) |  |
| ≥2 | 56 (51.4%) | 19 (67.9%) |  | 14 (51.9%) | 61 (55.5%) |  | 32 (49.2%) | 43 (59.7%) |  |
| Baseline lab |  |  |  |  |  |  |  |  |  |
| Neutrophil count,  cells/μL | 5102  (3807, 6341) | 7490  (6087, 8904) | <0.001 | 3847  (3556, 5856) | 5798  (4397, 7423) | 0.001 | 5394  (3768, 6579) | 5790  (4431, 7475) | 0.085 |
| Lymphocyte count,  cells/μL | 1721  (1441, 2086) | 1237  (948, 1451) | <0.001 | 2178  (1710, 2630) | 1534  (1296, 1837) | <0.001 | 1630  (1332, 2161) | 1570  (1360 ,1968) | 0.666 |
| Monocyte count,  cells/μL | 629 (501, 780) | 706 (555, 933) | 0.103 | 482 (416, 576) | 682 (550, 820) | <0.001 | 572 (466, 684) | 709 (571, 860) | <0.001 |
| Platelet count,  10^3^ cells/μL | 241 (176, 282) | 225 (202, 276) | 0.827 | 246 (189, 290) | 235 (181, 280) | 0.321 | 230 (187, 276) | 246 (183, 291) | 0.533 |
| Total bilirubin, mg/dL | 0.7 (0.5, 0.9) | 0.8 (0.6, 1.2) | 0.268 | 0.7 (0.5, 0.9) | 0.7 (0.6, 0.9) | 0.691 | 0.7 (0.5, 0.8) | 0.8 (0.6, 1.0) | 0.046 |
| ALP, IU/L | 133 (93, 222) | 185 (131, 346) | 0.008 | 101 (87, 191) | 156 (104, 257) | 0.052 | 113 (87, 158) | 189 (129, 291) | <0.001 |
| Albumin, g/dL | 3.9 ± 0.4 | 3.6 ± 0.7 | 0.011 | 4.1 (4.0, 4.3) | 3.9 (3.5, 4.2) | 0.012 | 4.2 (4.0, 4.4) | 3.6 (3.4, 4.0) | <0.001 |
| CA 19-9, U/mL | 241  (36, 4280) | 369  (16, 5532) | 0.921 | 141 (38, 6530) | 271 (23, 3324) | 0.914 | 266 (20, 1200) | 275 (32, 845) | 0.284 |
| Abbreviations: NLR, neutrophil-to-lymphocyte ratio; LMR, lymphocyte-to-monocyte ratio; AGR, albumin-to-globulin ratio; BMI, body mass index; ECOG PS, Eastern Cooperative Oncology Group performance status; CCI, Charlson comorbidity index; ALP, alkaline phosphatase; CA 19-9, carbohydrate antigen 19-9 | | | | | | | | | |

| **Supplementary Table 2. Multivariable Cox regression analysis of factors associated with progression-free survival (SIR marker-specific model).** | | | | | | |
| --- | --- | --- | --- | --- | --- | --- |
| **Variable** | **Model 1 (PLR)** | | **Model 2 (NLR)** | | **Model 3 (LMR)** | |
|  | **HR** | ***P* value*** | **HR** | ***P* value†** | **HR** | ***P* value‡** |
| PLR |  | **0.009** |  |  |  |  |
| ≤148 | 1 |  |  |  |  |  |
| >148 | 1.766 (1.155-2.703) |  |  |  |  |  |
| NLR |  |  |  | **0.052** |  |  |
| ≤5 |  |  | 1 |  |  |  |
| >5 |  |  | 1.638 (0.995-2.696) |  |  |  |
| LMR |  |  |  |  |  | **0.070** |
| <3.5 |  |  |  |  | 1 |  |
| ≥3.5 |  |  |  |  | 1.583 (0.963-2.603) |  |
| Abbreviations: SIR, systemic inflammatory response; PLR, platelet-to-lymphocyte ratio; NLR, neutrophil-to-lymphocyte ratio; LMR, lymphocyte-to-monocyte ratio  Number of metastatic sites and albumin were not included in all models because of their collinearity with SIR marker.  *Adjusted by distant lymph node metastasis and PLR  **†** Adjusted by distant lymph node metastasis and NLR  **‡** Adjusted by distant lymph node metastasis and LMR | | | | | | |

| **Supplementary Table 3. Multivariable Cox regression analysis of factors associated with overall survival (SIR marker-specific model).** | | | | | | | | |
| --- | --- | --- | --- | --- | --- | --- | --- | --- |
| **Variable** | **Model 1 (PLR)** |  | **Model 2 (NLR)** | | **Model 3 (LMR)** | | **Model 4 (AGR)** | |
|  | **HR** | ***P* value*** | **HR** | ***P* value†** | **HR** | ***P* value‡** | **HR** | ***P* value§** |
| PLR |  | **<0.001** |  |  |  |  |  |  |
| ≤148 | 1 |  |  |  |  |  |  |  |
| >148 | 2.182 (1.512-3.150) |  |  |  |  |  |  |  |
| NLR |  |  |  | **0.027** |  |  |  |  |
| ≤5 |  |  | 1 |  |  |  |  |  |
| >5 |  |  | 1.714 (1.063-2.762) |  |  |  |  |  |
| LMR |  |  |  |  |  | **0.001** |  |  |
| <3.5 |  |  |  |  | 1 |  |  |  |
| ≥3.5 |  |  |  |  | 2.199 (1.367-3.538) |  |  |  |
| AGR |  |  |  |  |  |  |  | 0.293 |
| <1.2 |  |  |  |  |  |  | 1 |  |
| ≥1.2 |  |  |  |  |  |  | 1.238 (0.832-1.844) |  |
| Abbreviations: SIR, systemic inflammatory response; NLR, neutrophil-to-lymphocyte ratio; LMR, lymphocyte-to-monocyte ratio; AGR, albumin-to-globulin ratio; ECOG PS, Eastern Cooperative Oncology Group performance status  Number of metastatic sites and albumin were not included in all models because of their collinearity with SIR marker.  *Adjusted by ECOG PS, distant lymph node metastasis, alkaline phosphatase, and PLR.  †Adjusted by ECOG PS, distant lymph node metastasis, alkaline phosphatase, and NLR.  ‡Adjusted by ECOG PS, distant lymph node metastasis, alkaline phosphatase, and LMR  §Adjusted by ECOG PS, distant lymph node metastasis, alkaline phosphatase, and AGR | | | | | | | | |
